# Development of simplex and multiplex immunochromatographic assays for rapid detection of GES-, PER-, and VEB-producing gram-negative bacteria

**DOI:** 10.1128/spectrum.01544-26

**Published:** 2026-09-11

**Authors:** Melisa Nicević, Christian Moguet, Saoussen Oueslati, Stéphanie Gelhaye, Arnaud Chalin, Marie Favé, Anaïs Vogel, Maid Rifatbegović, Laurent Dortet, Hervé Volland, Thierry Naas

**Affiliations:** 1 Team ‘Resist’, UMR1184 ‘Infectious Disease Models and Innovative Therapies (IDMIT)’ INSERM, Faculty of Medicine, Université Paris-Saclay, CEA, Healthi 89691, Le Kremlin-Bicêtre, France; 2 Department of Pathobiology and Epidemiology, University of Sarajevo – Veterinary Faculty 112354, Sarajevo, Bosnia and Herzegovina; 3 Département Médicaments et Technologies pour la Santé, Université Paris-Saclay, CEA, INRAE 27048, Gif-sur-Yvette, France; 4 Research and Development Department, NG Biotech, Guipry, France; 5 Department of Bacteriology-Hygiene, Bicêtre Hospital, APHP Paris-Saclay, Le Kremlin-Bicêtre, France; 6 SEPSIS Comprehensive Center – IHU SEPSIS 851435 , Garches, France; 7 French National Reference Center for Antibiotic Resistance: Carbapenem-Resistant Enterobacterales, Le Kremlin-Bicêtre, France; JMI Laboratories, North Liberty, Iowa, USA

**Keywords:** detection, ESBLs, LFIA

## Abstract

Extended-spectrum β-lactamase (ESBL)-producing *Enterobacteriaceae* are increasingly reported worldwide, emphasizing the need for rapid diagnostic tools to limit their spread and prevent outbreaks. Although CTX-M enzymes remain predominant, less common ESBLs such as GES, PER, and VEB are emerging and gaining clinical relevance. We developed simplex and multiplex lateral flow immunoassays (LFIAs) for the rapid detection of these ESBLs. Monoclonal antibodies were produced and incorporated into dedicated LFIA strips. The assays were evaluated using 116 WGS-characterized gram-negative bacteria (GNB) isolates expressing diverse β-lactamases and grown on various media. For testing, three colonies were suspended in 150 µL of extraction buffer, applied to the strips, and results were visually interpreted after 15 min. Both simplex and multiplex LFIAs achieved 100% sensitivity and specificity for detecting GES-, PER-, and VEB-producing isolates, irrespective of bacterial species, β-lactamase content, or growth conditions. The panel included prevalent variants of PER (*n* = 5, including PER-2), VEB (*n* = 9), and GES (*n* = 9, including ESBLs and carbapenemases). Detection limits for recombinant PER-1, VEB-1, and GES-1 were 5, 50, and 100 pg/mL, respectively, and 10^5^, 10^6^, and 10^6^ CFU/mL for corresponding *Acinetobacter baumannii* producers grown in tryptic soy broth. Overall, these simplex and multiplex GES-, PER-, and VEB-targeting LFIAs provide a rapid, accurate, and user-friendly approach suitable for routine use in clinical microbiology laboratories. When combined with the NG-Test CTX-M MULTI assay, which detects the five major CTX-M groups, they may further enhance the overall detection of the most common ESBLs, particularly among non-fermenting GNB.

**IMPORTANCE:**

Although less prevalent globally than CTX-M-type ESBLs, GES-, PER-, and VEB-type β-lactamases represent an important and often underrecognized mechanism of resistance, particularly in non-fermenting gram-negative pathogens. These enzymes are frequently associated with multidrug-resistant organisms and may significantly compromise available therapeutic options. The detection of the genes encoding these enzymes is often based on homemade PCR. Rapid diagnostic tests based on lateral flow immunoassays (LFIAs) have been successfully used to detect CTX-M-like ESBLs and the five main carbapenemases. Here, we demonstrated that GES, PER, and VEB enzymes can be readily detected by simplex or multiplex LFIAs from Enterobacterales, *Pseudomonas aeruginosa*, and *Acinetobacter baumannii* colonies. These assays are rapid (≤15 min), highly sensitive (10^5^–10^6^ CFU/mL), requiring no specialized equipment, thus offering a user-friendly alternative to molecular assays in clinical laboratories. These features align well with the World Health Organization ASSURED criteria (Affordable, Sensitive, Specific, User-friendly, Rapid, Equipment-free, and Deliverable).

## INTRODUCTION

Among gram-negative bacteria (GNB), extended-spectrum β-lactamase (ESBL)-producing Enterobacterales have emerged as a major clinical concern, as they are often susceptible only to a limited number of last-resort antibiotics, including carbapenems and newer β-lactam/β-lactamase inhibitor combinations (1, 2). Invasive infections caused by ESBL-producing *Escherichia coli* and *Klebsiella pneumoniae* are associated with higher rates of treatment failure, prolonged hospital stays, and increased mortality compared with infections caused by susceptible strains (3, 4). TEM- and SHV-type ESBLs were historically among the most prevalent ESBLs worldwide, particularly in hospital-associated Enterobacterales. However, their prevalence has declined in many regions following the global expansion of CTX-M enzymes, which now account for the majority of ESBL-mediated resistance worldwide (2, 5–7). In contrast, PER-, VEB-, and GES-type ESBLs are considered “minor ESBLs” because they remain comparatively uncommon and have traditionally been associated with localized outbreaks or specific geographic regions. Nevertheless, these enzymes are clinically important due to their frequent association with multidrug resistance and, in the case of some GES variants, carbapenemase activity. Moreover, they are being increasingly reported worldwide in clinical GNB, including *Pseudomonas aeruginosa*, *Acinetobacter baumannii*, and *Enterobacteriaceae* (6–8).

ESBLs classically hydrolyze expanded-spectrum cephalosporins (ESCs), spare carbapenems, and are inhibited by clavulanic acid. Consequently, carbapenems are frequently used as first-line therapy, increasing selective pressure and contributing to the emergence of carbapenem-resistant organisms (9).

GES-1 (Guiana Extended-Spectrum) was initially described from a *K. pneumoniae* isolate from French Guiana (10) and has since rapidly spread globally, best exemplified by the increasing number of GES variants described in the ß-lactamase database (BLDB) (*n* = 69; http://www.bldb.eu [8]), differing by 1–16 amino-acid (AA) substitutions (Fig. S1) and their spread in humans, animals, and the environment (7, 11–13). Among the 67 GES variants for which the AA sequences are available, 43 (64.2%) with either Gly170Asn (*n* = 3; 4.5%) or Gly170Ser (*n* = 40; 59.7%) substitutions have carbapenem-hydrolytic activity, while the others are considered classical ESBLs without any carbapenemase activity (7, 8, 11, 13). Numerous outbreaks on all continents with GES-producing isolates have been described in the literature (14–19). GES-type enzymes are usually plasmid-encoded and located within the variable region of class 1 integrons, thereby facilitating horizontal gene transfer and promoting their dissemination among Enterobacterales as well as non-fermenting GNB.

PER-1 (Pseudomonas Extended Resistance) initially described in *P. aeruginosa* from a Turkish patient in 1993, has since been identified in a wide range of GNB, including *Acinetobacter* spp. and Enterobacterales (7, 20, 21). PER-type enzymes are usually plasmid-encoded, facilitating horizontal gene transfer, and their spread has been reported mainly in the Mediterranean region, the Middle East, and parts of Asia (7, 19, 20). PER-2, a closely related variant (85% AA identity with PER-1), has been predominantly reported in Enterobacterales—especially *E. coli* and *K. pneumoniae*—with a notable geographic distribution in South America (22). Eighteen PER variants, belonging to three PER subfamilies: PER-1 (*n* = 12 variants), PER-2 (*n* = 3 variants displaying 84% AA identity with PER-1), and PER-12 (*n* = 3 variants displaying 94% AA identity with PER-1), have been described in the BLDB (Fig. S2) (8).

VEB (Vietnamese Extended-spectrum β-lactamase)-type ESBLs initially identified in an *E. coli* isolate from Vietnam, have since been reported in a variety of GNB, including *P. aeruginosa*, *Acinetobacter* spp., and Enterobacterales (7, 20, 23–25). VEB β-lactamases are often associated with integrons and plasmids, facilitating their dissemination across bacterial species (7). Forty-two VEB variants differing by 1–7 AA (Fig. S3) have been described in the BLDB (*n* = 42) (8). To date, there have been reports of nationwide outbreaks involving the VEB-1 producers from several continents (7, 20, 26–29).

Although less prevalent globally than CTX-M-type ESBLs, PER-, VEB-, and GES-type β-lactamases represent an important and often underrecognized mechanism of resistance, particularly in non-fermenting gram-negative pathogens. They are often associated with multidrug-resistant pathogens and may significantly impair available therapeutic options. GES-type β-lactamases are probably the most worrisome, as they can extend their spectra of hydrolysis toward carbapenems by single-point mutations (7, 11). Although cefiderocol is generally stable against most ESBLs, elevated MICs have been reported in isolates producing PER- and VEB-type enzymes, particularly in *P. aeruginosa* and *Acinetobacter* spp. (30–32). This reduced activity is likely multifactorial, resulting from the combined effect of these ESBLs with additional resistance mechanisms such as porin loss or efflux pump overexpression (31). In contrast, taniborbactam, a novel broad-spectrum β-lactamase inhibitor, has demonstrated *in vitro* inhibitory activity against class A ESBLs, including PER-, VEB-, and GES-type enzymes, restoring the activity of partner β-lactams (31). These findings highlight the importance of detecting these non-CTX-M ESBLs to initiate proper treatment.

Phenotypic detection of non-CTX-M ESBLs such as PER-, VEB-, and GES-type enzymes remains challenging, as routine ESBL screening methods are primarily optimized for TEM, SHV, and CTX-M producers. These enzymes often confer high-level resistance to ceftazidime, sometimes exceeding that to cefotaxime, and may display atypical synergy patterns in clavulanate-based confirmatory tests (7). In addition, the frequent coexistence of other resistance mechanisms—such as AmpC overproduction, porin loss, or efflux—can mask ESBL activity and complicate phenotypic interpretation. As a result, reliable identification of PER-, VEB-, and GES-type enzymes often requires complementary molecular methods.

Homemade polymerase chain reaction (PCR)-based methods targeting specific *bla*_GES_, *bla*_PER_, and *bla*_VEB_ genes are widely used during outbreaks or in areas where they are more prevalent and allow rapid and sensitive identification of these genes, irrespective of whether they are expressed or not (7, 33). These assays can be implemented as simplex or multiplex formats and are well suited for both Enterobacterales and non-fermenting GNB (7). Only a few PCR assays are commercially available, such as Amplidiag CarbaR + MCR Kit (Mobidiag, Paris, France) that detects *bla*_GES_ genes (34) and EasyScreen ESBL/CPO Detection Kit (Genetic Signature, Sydney, Australia) that detects *bla*_GES_, *bla*_PER_, and *bla*_VEB_ genes (35). Whole-genome sequencing further enables comprehensive characterization, including discrimination among enzyme variants and detection of coexisting resistance determinants (36). Despite their accuracy, molecular methods remain limited by cost, infrastructure requirements, and turnaround time, restricting their routine use in many clinical microbiology laboratories (34). Rapid diagnostic tests based on lateral flow immunoassays (LFIAs) have been successfully used to detect CTX-M-like ESBLs (NG-Test CTX-M MULTI; NG Biotech, Guipry, France), the five main carbapenemases (NG-Test CARBA 5), and recently GES enzymes (NG-Test GES) (37–40). These successes suggest that LFIA could be extended to detect PER- and VEB-type ESBLs, offering a rapid, user-friendly alternative to molecular assays in clinical laboratories.

The aim of this study was to develop and evaluate the analytical performance of simplex and multiplex LFIA assays capable of detecting GES, VEB, and PER β-lactamases in GNB.

## MATERIALS AND METHODS

### β-lactamase purification

The *bla*_VEB-1_ and *bla*_PER-1_ gene fragments corresponding to the mature β-lactamases were cloned into the expression vector pET41b (+) (Novagen, VWR International, Fontenay-sous-Bois, France) (41). VEB-1 and PER-1 were expressed and purified with the hexa-histidine tag on its C-terminus in one-step pseudo-affinity chromatography using an NTA-Nickel column (GE Healthcare, Les Ulis, France) as previously described (41).

### Monoclonal antibodies (mAbs) and assay development

Biozzi mice (10 weeks old) were immunized with the recombinant PER-1 and VEB-1 β-lactamase as previously described (39, 40). Twenty monoclonal antibodies (mAbs) from each immunization were tested, and the pairs of antibodies showing the best limit of detection (LOD) on PER-1 and PER-2 and on VEB-1-expressing bacteria were selected and subsequently produced on a large scale. These anti-PER and anti-VEB antibodies, together with those targeting GES, previously selected and characterized using GES-5 recombinant protein (40), were used for the development of the simplex and multiplex assay as previously described (39).

**Fig 1:**
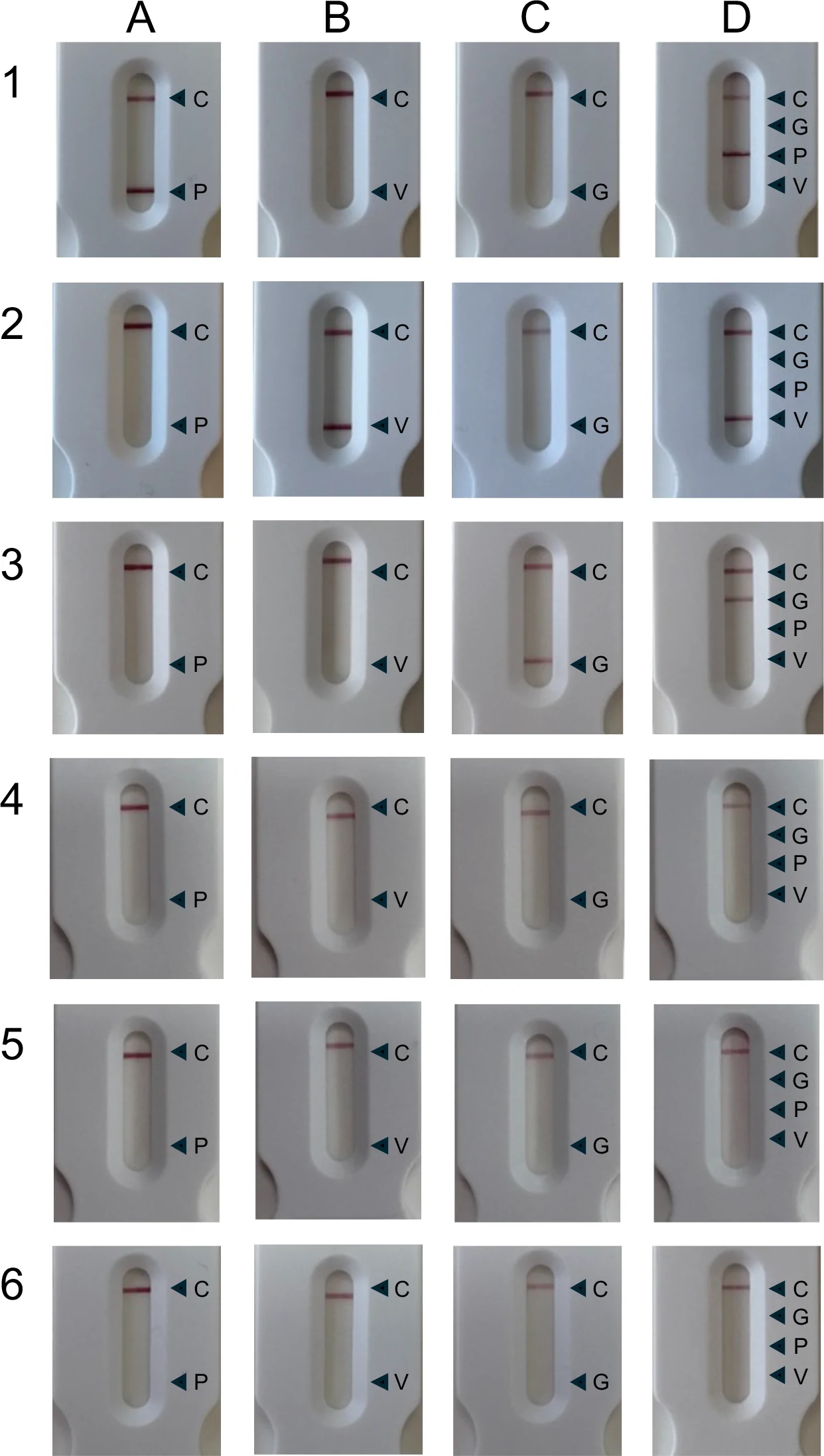
Example of LFIA results for (1) *A. baumannii* O23D4 producing PER-2, (2) *K. pneumoniae* 579A1 producing VEB-1, (3) *P. aeruginosa* Qia-21 producing GES-9, (4) *E. coli* 2H6 producing CTX-M-15, (5) *P. aeruginosa* R7F1 producing SHV-5, and (6) *A. baumannii R8F9* producing SHV-5, obtained with simplex (**A**) PER LFIA, (**B**) VEB LFIA, (**C**) GES LFIA, and multiplex (**D**) multi GPV LFIA. “C” indicates the control line, “P” PER-specific test line, “V” VEB-specific test line, and “G” GES-specific test line.

**Fig 2:**
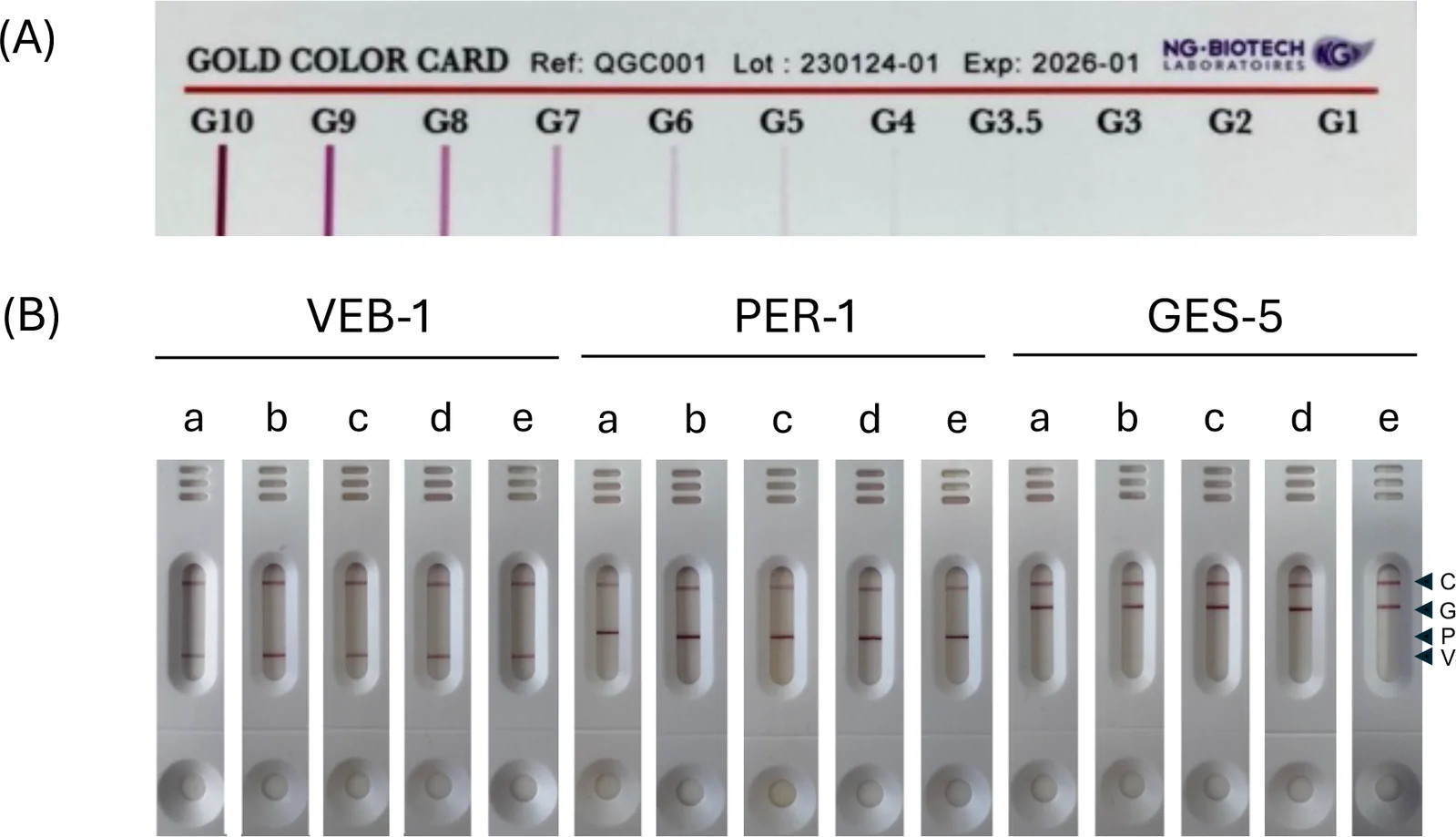
Multiplex GES/PER/VEB LFIA (multi GPV LFIA) test results from bacterial isolates grown on different culture media. (**A**) Intensity ruler from NG-Biotech. After 15 min of incubation, the intensity of each band was visually compared to the intensity ruler and scored accordingly. (**B**) Multi GPV LFIA test results for VEB-1-producing *E. coli* 569B8, PER-1-producing *A. baumannii* Ceph123, and GES-5-producing *P. aeruginosa* O58B2 on different culture media: (a) chromogenic and selective ChromID ESBL agar (bioMérieux); (b) Drigalski agar (bioMérieux); (c) Mueller Hinton agar (bioMérieux); (d) Columbia agar plus 5% horse blood (bioMérieux); and (e) UriSelect 4 agar (Bio-Rad). Pictures were taken after 15 min of migration.

Three isolated colonies were touched and resuspended in five drops (*circa* 150 µL) of the extraction buffer and vortexed for a few seconds. Subsequently, 100 µL of this extract was dispensed onto the LFIA cassettes, and the migration was followed for 15 min. The results were read by eye by monitoring for the appearance of a red band on the test line, along with a band corresponding to the control line (Fig. 1). Time to positivity and the intensity of the test line after 15 min of incubation were recorded using an intensity ruler (Fig. 2A).

### Limit of detection (LOD) using recombinant proteins and bacterial extracts

Serial dilutions of the recombinant proteins used for immunization (50,000–0 pg/mL) was performed with extraction buffer. Similarly, GES-11-producing *A. baumannii* G2O56 (42), PER-1-producing *A. baumannii* Ceph 123 (43), and VEB-1-producing *A. baumannii* AYE (29) were serially diluted in Tryptic Soy Broth (bioMérieux, Marcy l’Etoile, France) (10^8^, 1.2 × 10^8^, 2.4 × 10^7^, 4.8 × 10^6^, 9.6 × 10^5^, and 1.9 × 10^5^ CFU/mL). Bacterial dilution vials were centrifuged for 20 min at 10,000 × *g*; the supernatant was carefully discarded, and the pellet was resuspended in the same volume of extraction buffer as initial TSB. Consecutively, 100 μL of serially diluted proteins and of bacterial extracts were dispensed onto the cassette, and results were read by eye after 15 min.

### Bacterial strains

One hundred and sixteen whole-genome-sequenced GNB isolates were used to evaluate the simplex and multiplex LFIAs. This collection included 77 *Enterobacteriaceae* (19 *E. coli*, 17 *K. pneumoniae*, 12 *Morganella* spp., 9 *Citrobacter freundii*, 9 *E. cloacae* complex, 5 *Proteus mirabilis*, 2 *Providencia stuartii*, 3 *Serratia marcescens*, and 1 *Salmonella* Enteritidis), 19 *Pseudomonas* spp. (18 *P. aeruginosa* and 1 *P. guariconensis),* and 20 *Acinetobacter* spp. (16 *A. baumannii*, 1 *A. baylyi*, 1 A. *ursingii*, 1 *A. johnsonii*, and 1 *A. lwolfii*) isolates, harboring single or multiple β-lactamase genes, including carbapenemases, ESBLs, and plasmid- or chromosome-encoded AmpCs grown on URISelect 4 (Uri4; Bio-Rad, Marne-la-Coquette, France) plates. Among these isolates, 21 produced GES-variants corresponding to 9 out of 69 known alleles, (namely GES-1, -2, -5, -6, -7, -9, -11, -12, and -14 alleles), 20 produced PER-variants corresponding to 5/18 alleles (namely PER-1, -2, -3, -6, and -7), and 21 produced VEB-variants corresponding to 8/42 alleles (namely VEB-1, -5, -6, -9, -14, -25, -36, and -37) (Table 1 and Fig. S1 to S3).

**TABLE 1:** β-lactamase content and LFIA results of bacterial isolates tested

	Acquired β-lactamases	# of isolates	GES LFIA results	PER LFIA results	VEB LFIA results	Multi GPV^*a*^ LFIA results
*Enterobacteriaceae* (*n* = 77)						
GES-ESBLs	GES-1 (*n* = 3); GES-7 (*n* = 2)	5	5^*b*^	–^*c*^	–	5
GES-carbapenemase	GES-5 (*n* = 3); GES-6	4	4	–	–	4
VEB	VEB-1 (*n* = 7), VEB-5, VEB-6 (*n* = 3), VEB-9, VEB-14 (*n* = 2), VEB-25, VEB-36, VEB-37	17	–	–	17	17
PER	PER-1, PER-2 (*n* = 2), PER-3, PER-6	5	–	5	–	5
WT	*E. coli*, *S. marcescens*, *C. freundii* (*n* = 2), *K. pneumoniae*, *K. oxytoca*, *P. mirabilis*, *Morganella* sp. (*n* = 10)	17	–	–	–	–
Other ESBLs	BEL-1; CTX-M-1, CTX-M-2, CTX-M-3, CTX-M-8, CTX-M-14, CTX-M-15 (*n* = 2), CTX-M-25; TEM-24	10	–	–	–	–
Carbapenemase	NDM-5; OXA-48-like; IMI-1; SME-1; GIM-1 (*n* = 2), KPC-2 (*n* = 2); VIM-1, IMP-1	10	–	–	–	–
Other 3ESC^*d*^ resistances	Overexpressed Ampc *E. cloacae complex,* CMY (*n* = 4), DHA-2, SRT-2, ACC-1 (*n* = 2)	9	–	–	–	–
*Pseudomonas* spp. (*n* = 19)						
GES-ESBLs	GES-1; GES-9 (*n* = 2)	3	3	–	–	3
GES-carbapenemases	GES-2; GES-5; GES-14	3	3	–	–	3
VEB	VEB-1 (*n* = 2), VEB-9	3	–	–	3	3
PER	PER-1 (*n* = 3), PER-3	4	–	4	–	4
WT	*P. aeruginosa*	1	–	–	–	–
Other ESBLs	SHV-2; BEL-2	2	–	–	–	–
Other ESC resistance	GIM-1; CMY/DHA, VIM-2	3	–	–	–	–
*Acinetobacter* spp. (*n* = 20)						
GES-ESBLs	GES-1, GES-11, GES-12 (*n* = 2)	4	4	–	–	4
GES-carbapenemases	GES-14	1	1	–	–	1
VEB	VEB-1	1	–	–	1	1
PER	PER-1 (*n* = 5), PER-2 (*n* = 5), PER-7	11	–	11	–	11
Other ESBL	SHV-5	1	–	–	–	–
Other ESC resistance	VIM-4	1	–	–	–	–
WT	*A. baumannii* WT	1	–	–	–	–
Total		116	20	20	21	61
Test performance						
Specificity^*e*^			100**%** (CI = 83.16–100%)	100**%** (CI = 83.16–100%)	100**%** (CI = 83.89–100%)	100**%** (CI = 94.13–100%)
Sensitivity^*e*^			100**%** (CI = 96.23–100%)	100**%** (CI = 96.23–100%)	100**%** (CI = 96.1–100%)	100**%** (CI = 93.51–100%)
Youden’s Index^*f*^			100	100	100	100

^*a*^ Multi GPV LFIA: multiplex GES/PER/VEB LFIA.

^*b*^ Number of isolates that gave a positive test band.

^*c*^ – indicates negative test result for tested isolates.

^*d*^ ESC, expanded-spectrum cephalosporin.

^*e*^ The sensitivities and specificities were calculated with their respective 95% confidence interval (95% CI) using the free software MedCalc: Website for statistical computation at https://www.medcalc.org/en/calc/diagnostic_test.php. The gold standard was WGS confirmed presence of resistance determinants. An ESBL prevalence rate of 10%, corresponding to the French prevalence, was applied.

^*f*^ The Youden’s index (sensitivity (%) + specificity (%) − 100) calculates the efficiency of a diagnostic test; a Youden score of 100 indicates a perfect test.

### Culture media tested

Three representative isolates (VEB-1-producing *E. coli* 569B8, GES-5-producing *P. aeruginosa* O58B2, and PER-1-producing *A. baumannii* Ceph 123) were grown on five commonly used culture media: URISelect 4 (Bio-Rad), Muller-Hinton agar, Columbia agar plus 5% horse blood, ChromID ESBL agar, and Drigalski agar (bioMérieux) to test possible interference of LFIAs with culture media.

### Statistical analysis

The 95% confidence intervals (95% CIs) were calculated using the online MedCalc statistical software (https://www.medcalc.org/calc/diagnostic_test.php). Box plots were generated using Excel 2026 (Microsoft, Paris, France).

## RESULTS

### Determination of the limits of detection

The limits of detection (LODs) for GES, PER, and VEB were identical for the simplex and multiplex strips, corresponding to 100, 4.3, and 50 pg/mL of the respective recombinant GES, PER, and VEB proteins. Similarly, using *A. baumannii* isolates expressing GES, PER, or VEB, the LODs obtained with both the simplex and multiplex strips were 10^6^, 10^5^, and 10^6^ CFU/mL, respectively.

### Evaluation of simplex LFIAs with isolates grown on UriSelect 4 media

Simplex LFIA assays were able to detect all the GES, PER, and VEB variants irrespective of the bacterial species tested, with 100% sensitivity and specificity, using pure isolates grown on URISelect 4 plates (Bio-Rad) (Table 1 and Fig. 1 [1–3]). Twenty GES-producers expressing 9 of the 69 known variants (namely GES-1, -2, -5, -6, -7, -9, -11, -12, and -14 alleles), 20 PER-producers expressing 5 of the 18 known variants (namely PER-1, -2, -3, -6, and -7), and 21 VEB-producers expressing 8 of the 42 known variants (namely VEB-1, -5, -6, -9, -14, -25, -36, and -37) were tested. These alleles correspond to the most prevalent variants (7). Wild-type isolates or those expressing non-targeted β-lactamases, including cephalosporinases, other ESBLs, or carbapenemases, gave negative results in every species tested (Table 1 and Fig. 1 [4–6]).

**Fig 3:**
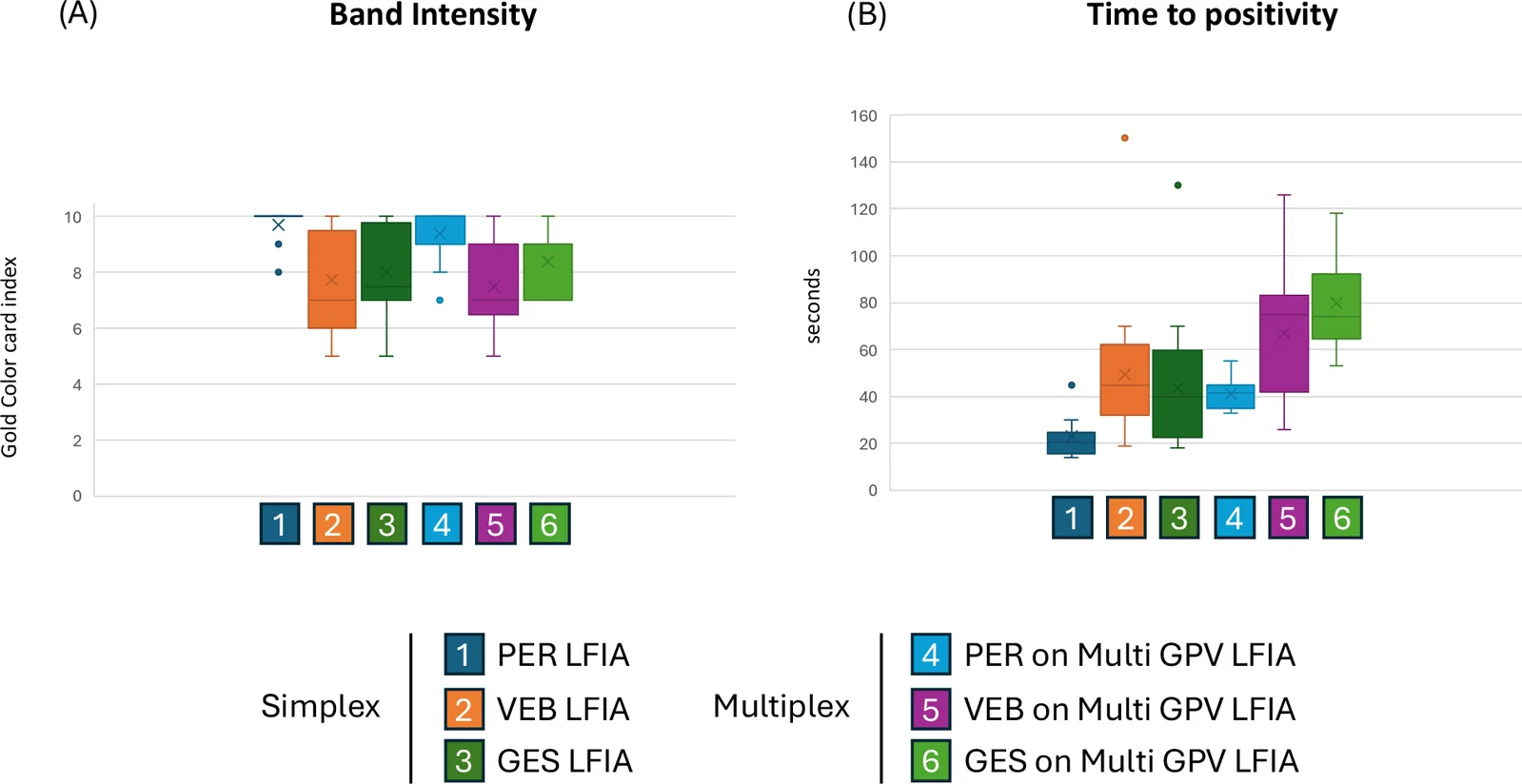
Boxplot of (**A**) intensity of bands at 15 min reading according to intensity ruler from NG Biotech (Fig. 2A), going from G1 (hardly visible by eye) to G10 (very intense), and (**B**) time (seconds) for band appearance using simplex PER, GES, and VEB LFIAs and multi GPV LFIA (GES-PER-VEB).

The band intensities after 15 min of migration were strong according to the NG-Biotech intensity ruler (Fig. 2A), with intensities on average of 8, 9.7, and 7.7 for GES, PER, and VEB LFIAs, respectively, thus allowing easy reading of the signal (Fig. 2A and 3). The antibodies directed against PER enzymes seemed to give stronger and more uniform signals, even with PER-2, which shares only 84% AA identity with PER-1. The time to positivity was very rapid. On average, it took 44, 23, and 49 s for GES-, PER-, and VEB-specific bands to appear (Fig. 3).

### Evaluation of multiplex LFIAs with isolates grown on UriSelect 4 media

The multiplex LFIA assay combining all the antibodies that detect the three minor ESBLs detected all the GES-, VEB-, and PER-expressing GNB irrespective of the variants and the bacterial species tested [Table 1 and Fig. 1 (1–3)]. The sensitivity and specificity were 100% using pure isolates grown on UriSelect 4 plates. No cross-reactivity was observed among the three pairs of antibodies in the multiplex configuration. Differences were observed among band intensities, but they were all strong enough to be easily read by eye. On average, band intensities of 8.4, 9.4, and 7.5 were recorded for GES, PER, and VEB bands, respectively (Fig. 3). Although the time to positivity was very rapid, it took slightly longer compared to simplex assays, which is probably due to the longer distances of migration on the multiplex strip. On average, it took 80, 41, and 66 s for the GES-, PER-, and VEB-specific bands to appear (Fig. 3).

All the isolates expressing non-targeted β-lactamases, such as cephalosporinases, other ESBLs, or carbapenemases, gave negative results irrespective of the species tested (Enterobacterales, *P. aeruginosa,* and *A. baumannii*) (Table 1 and Fig. 1 [4–6]).

### Evaluation of simplex and multiplex LFIAs with isolates grown on different culture media

Three representative isolates (VEB-1-producing *E. coli* 569B8, GES-5-producing *P. aeruginosa* O58B2, and PER-1-producing *A. baumannii* Ceph 123) were grown on five media classically used for bacterial growth (Table S1 and Fig. 2), including two chromogenic media, one used for bacterial identification (UriSelect 4), and one used for the selection of ESBL-expressing strains (ChromID ESBL), which generate colonies with genus-specific colors. These colored colonies, once suspended in the extraction buffer, stained the buffer in a similar manner, but the staining did not interfere with the migration, nor with the band appearance on the nitrocellulose membrane, and still yielded easily interpretable results (Fig. 2B). The GES-, PER-, and VEB-expressing strains gave positive results on the respective simplex strips (Table S1) and on the specific band on the multiplex strip (Fig. 2B), while other ESBL-producers with CTX-M-15 or SHV-5 gave negative results irrespective of the tested species and on all tested strips.

## DISCUSSION

The production of ESBLs among *Enterobacteriaceae*, *Pseudomonas* spp., and *Acinetobacter* spp. has become a noteworthy mechanism for multidrug resistance. The rapid and effective detection of antibiotic-resistant bacteria is a critical step for antibiotic stewardship and infection control (2, 3). Despite technological improvements, the identification of pathogenic bacteria, as well as the detection of antibiotic resistance, remains complex and time-consuming, with time to results often over 24 h, or very costly for molecular assays (33, 44). Lateral flow immunoassays (LFIAs) have proven to be useful as easy, rapid, and reliable confirmatory tests for detection of CTX-M ESBLs in gram-negatives, which now account for the vast majority of ESBLs in Enterobacterales worldwide (2, 5). However, minor ESBLs of PER-, VEB-, and GES-type are increasingly isolated worldwide in clinical GNB, especially in *P. aeruginosa* and *A. baumannii* (7, 8). As they are not detected by commercially available diagnostic tests, they silently spread within healthcare settings and result in difficult-to-control outbreaks that are increasingly described globally (7, 14–20, 24, 40, 45).

In France, GES, PER, and VEB ESBLs are still rare in Enterobacterales. Indeed, during the period of 2023–2025, on average, one PER- and 10 VEB-producing Enterobacterales were received by the French National Reference Center for Antimicrobial Resistance (F-NRC-AMR) for carbapenem-resistant Enterobacterales (out of 6,000 isolates received per year), but the number of GES-producers rose from 36 isolates in 2023, 50 in 2024 to 70 isolates in 2025 (T. Naas, pers. comm.). In *A. baumannii*, 5 GES-11-producing and 6 PER-1-producing *A. baumannii* isolates (4% of total isolates) were received by the F-NRC-AMR in 2022 (46). However, in *P. aeruginosa*, a constant increase in the proportion of GES, PER, and VEB enzymes has been evidenced since 2016. PER-ESBLs represented the first class A ESBL (*n* = 34; 22%) in *P. aeruginosa* isolates from France in 2022 before GES-ESBLs (*n* = 31: 20%), SHV-ESBLs (*n* = 17: 11%), and VEB-1 (*n* = 14; 9%), but after OXA-ESBLs (*n* = 59, 38%) (46). Similarly, the proportion of GES-carbapenemase-producing *P. aeruginosa* in 2022 (*n* = 19, 11.2%) represented the third most prevalent carbapenemase in *P. aeruginosa*, after VIM (*n* = 96, 56%) and NDM (*n* = 33, 19%) carbapenemases (46).

Globally, GES-, PER-, and VEB-type ESBLs are considered relatively uncommon compared with the dominant CTX-M, TEM, and SHV families, although their prevalence has increased sporadically through regional outbreaks and clonal dissemination. Reviews of global ESBL epidemiology consistently describe these enzymes as “minor” or “less common” ESBLs, often associated with specific geographic hotspots and multidrug-resistant non-fermenters such as *P. aeruginosa* and *A. baumannii* (6). In a large systematic review covering studies published between 1990 and 2022, PER and VEB genes were reported in only approximately 2% of included studies, whereas CTX-M and, to a lesser extent, TEM and SHV enzymes were detected in the majority of reports (47).

GES enzymes have been reported worldwide, but their prevalence remains low overall, generally accounting for <1–5% of ESBL-producing Enterobacterales in most surveillance studies. Nevertheless, localized outbreaks with substantially higher frequencies have been described, particularly among *P. aeruginosa* and *A. baumannii*, and certain GES variants, such as GES-5, additionally exhibit carbapenemase activity (11, 13). Although the GES LFIA does not discriminate between GES-type ESBLs and GES carbapenemases, detection of either enzyme remains clinically important because GES enzymes are uncommon but can substantially influence antimicrobial susceptibility and guide therapeutic decisions (40, 48). Indeed, GES-ESBLs display low susceptibility to commercially available inhibitors (clavulanate and tazobactam), compromise ESC treatments, including cefepime, and they may evolve by point mutations *in vivo* upon antibiotic treatment with imipenem, cefotaxime, or aztreonam to become GES carbapenemases (48). Moreover, both GES-ESBLs and GES carbapenemases in *P. aeruginosa* have been associated with reduced susceptibility to ceftazidime-avibactam (46), underscoring the need for accurate MIC determination before treatment, and impaired activity of ceftolozane-tazobactam and cefiderocol (31, 49, 50). Therefore, their detection is likely to become increasingly clinically relevant in the coming years.

PER-type ESBLs are most commonly reported from Turkey, the Mediterranean region, South America, and parts of Asia, particularly in *Acinetobacter* and *Pseudomonas* species (6). In one study on 150 *A. baumannii* isolates from a Bolivian hospital, 106 carried *bla*_CTX-M-2_, 32 carried *bla*_CTX-M-43_, and 12 carried *bla*_PER-2_, corresponding to a prevalence of 8% for *bla*_PER-2_ (51). VEB-type ESBLs have been reported mainly in Southeast Asia, the Middle East, and France, often in *P. aeruginosa* and *A. baumannii*. Although global prevalence is low, VEB enzymes are clinically significant because they are frequently carried on integrons and multidrug-resistance plasmids that facilitate rapid dissemination in hospital settings (6, 7). PER and VEB enzymes confer resistance to broad-spectrum cephalosporins, including cefotaxime, ceftazidime, and cefepime, and are now reported worldwide with varying prevalence rates (7, 26). These enzymes pose a substantial threat to clinical therapy, especially for serious infections caused by *Pseudomonas* and *Acinetobacter* spp., as these strains are often multidrug-resistant, carrying other resistance genes such as carbapenemases or aminoglycoside-modifying enzymes. A recent study has highlighted that VEB-like and PER-like ESBLs may lead to cefepime-zidebactam resistance in *P. aeruginosa* recombinant strains (49). Detection of these enzymes in Enterobacterales has further expanded the clinical concern beyond non-fermenters. Indeed, the presence of PER and VEB β-lactamases has been involved in reduced susceptibility/resistance to newly released antibiotics, such as cefiderocol and aztreonam/avibactam (31).

Detection of GES, PER, and VEB enzymes is essential for antimicrobial resistance surveillance and infection control. Even though considered rare enzymes, they are spreading globally in both hospital and community settings, and their detection is crucial for epidemiological surveillance to identify emerging clones and resistance hotspots (7, 50). Furthermore, these ESBLs confer resistance to broad-spectrum β-lactams, including cephalosporins and monobactams, and are frequently carried on plasmids that also harbor other antimicrobial resistance genes, thereby promoting multidrug-resistant (MDR) phenotypes and limiting treatment options (2). Early identification may enable optimized therapy and help prevent treatment failures, especially in severe infections caused by MDR gram-negative bacilli (2). In several studies, LFIAs have been successfully applied directly to clinical specimens, including positive blood cultures, urine samples, and rectal swabs (37, 38, 52, 53). Given their high sensitivity, the GES-, PER-, and VEB-LFIAs could also be used on such matrices, allowing early detection of these resistance mechanisms.

The implementation of these LFIAs in clinical microbiology laboratories for the detection of GES, PER, and VEB ESBLs should be supported by a diagnostic stewardship program that considers both patient risk profiles and the local epidemiology of ESBLs, plasmid-mediated AmpC (pAmpC) β-lactamases, and carbapenemases. The local prevalence of these enzymes is a key determinant of their cost-effective use. In high-prevalence settings, such as Turkey for PER enzymes, Southeast Asia for VEB enzymes, and the Arabian Peninsula for GES enzymes, these assays may complement existing ESBL diagnostic tools, such as the NG-Test CTX-M MULTI, particularly for non-fermenting GNB, in which PER, VEB, and GES enzymes are more prevalent. Their implementation could therefore substantially improve the detection of ESBL-producing isolates that would otherwise remain undetected. Likewise, patients repatriated from these high-prevalence regions may be at increased risk of carriage or infection in otherwise low-prevalence settings. Finally, these assays may also be particularly useful for patients known to be colonized with these organisms, as 15–35% of them subsequently may develop infections caused by their colonizing strains (54).

### Conclusions

In this study, we demonstrated that GES, PER, and VEB enzymes can be readily detected by simplex or multiplex LFIAs in Enterobacterales, *P. aeruginosa*, and *A. baumannii*. The assays are rapid (≤15 min), highly sensitive (10^5^–10^6^ CFU/mL), user-friendly, and require no specialized equipment. They are compatible with most routine agar media and can be stored and performed at room temperature, making them suitable for laboratories with limited resources, including those in low- and middle-income countries. These features align well with the World Health Organization ASSURED criteria (Affordable, Sensitive, Specific, User-friendly, Rapid, Equipment-free, and Deliverable) (38, 55). Although the most prevalent variants were detected, larger panels should now be evaluated. However, given the conservation among GES and VEB enzymes and the successful detection of the distantly related PER-1 and PER-2 variants, it is likely that most known variants will be identified by these LFIAs.

The global spread of ESBLs, including GES, PER, and VEB, is facilitated by mobile genetic elements, such as plasmids, which allow for horizontal gene transfer among bacterial populations (56). Surveillance studies have documented the presence of these enzymes and their variants in numerous countries, highlighting the need for continuous monitoring and effective infection control measures (7). The clinical implications of these types of ESBLs are profound, as infections caused by these resistant organisms are associated with increased treatment failures, prolonged hospital stays, and higher healthcare costs (2).

## Data Availability

All data generated or analyzed during this study are included in this article. No additional data sets were generated or analyzed beyond those presented in this manuscript.

## SUPPLEMENTAL MATERIAL

Supplemental table and figures spectrum.01544-26-s0001.docx Table S1 and Fig. S1 to S3.

ASM does not own the copyrights to Supplemental Material that may be linked to, or accessed through, an article. The authors have granted ASM a non-exclusive, world-wide license to publish the Supplemental Material files. Please contact the corresponding author directly for reuse.
