# Supplementary material for: Development of simplex and multiplex immunochromatographic assays for rapid detection of GES-, PER-, and VEB-producing gram-negative bacteria
Source: Microbiol Spectr. 2026 Sep 11;14(10):e01544-26. doi: 10.1128/spectrum.01544-26 (PMC13688859; doi:10.1128/spectrum.01544-26)
Supplement: Supplementary file 1 [file spectrum.01544-26-s0001.docx]

**Supplemental material**

**Development of simplex and multiplex immunochromatographic assays for rapid detection of GES-, PER-, and VEB -producing Gram-negative bacteria*.***

Melisa NICEVIĆ,^1,2*^ Christian MOGUET,^3,4*^ Saoussen OUESLATI,^1,5*^ Stéphanie GELHAYE,^3^ Arnaud CHALIN,^4^ Marie FAVÉ,^3^ Anaïs VOGEL,^3^ Maid RIFATBEGOVIĆ,^2^ Laurent DORTET, ^1,5,6,7^ Hervé VOLLAND,^3,6^ Thierry NAAS^1,5,6,7#^

Table: 1

Figures: 3

**Table S1: GES/PER/VEB simplex and Multi GPV (GES-PER-VEB) LFIA results from bacterial isolates grown on different culture media**

|  |  | LFIA Test | Test results on culture medium^a^ | | | | |
| --- | --- | --- | --- | --- | --- | --- | --- |
| Bacterial species | β-lactamase |  | MH | COH | Uri-4 | DRIG | ChromID  ESBL |
| *A. baumannii*  (ceph123) | PER-1 | TL**^b^**-PER | **+^c^** | **+** | **+** | **+** | **+** |
|  |  | TL-GES | -^d^ | - | - | - | - |
|  |  | TL-VEB | - | - | - | - | - |
|  |  | TL-Multi | **+ PER** | **+ PER** | **+ PER** | **+ PER** | **+ PER** |
| *P. aeruginosa*  (O58B2) | GES-5 | TL-PER | - | - | - | - | - |
|  |  | TL-GES | **+** | **+** | **+** | **+** | **+** |
|  |  | TL-VEB | - | - | - | - | - |
|  |  | TL-Multi | **+ GES** | **+ GES** | **+ GES** | **+ GES** | **+ GES** |
| *E. coli*  (569B8) | VEB-1 | TL-PER | - | - | - | - | - |
|  |  | TL-GES | - | - | - | - | - |
|  |  | TL-VEB | **+** | **+** | **+** | **+** | **+** |
|  |  | TL-Multi | **+ VEB** | **+ VEB** | **+ VEB** | **+ VEB** | **+ VEB** |

^a^MH, Mueller-Hinton agar; COH, Columbia agar plus 5% horse blood; Uri-4, URISelect 4 medium; DRIG, Drigalski agar; ChromID, ChromID^TM^ ESBL; ^b^ TL: test line; ^c^+, positive LFIA result; ^d^-, negative LFIA result.

Figure S1: Phylogeny of GES variants present in the BLDB^1^. Multiple alignment was done using ClustalW and tree was calculated using PhyML v20160115 (<https://www.genome.jp/tools-bin/clustalw>). % AA identities with GES-1 are indicated. Red boxes indicate tested variants.

Figure S2: Phylogeny of PER variants present in the BLDB^1^. Multiple alignment was done using ClustalW and tree was calculated using PhyML v20160115 (<https://www.genome.jp/tools-bin/clustalw>). % AA identities with PER-1 are indicated. Red boxes indicate tested variants.

Figure S3: Phylogeny of VEB variants present in the BLDB^1^. Multiple alignment was done using ClustalW and tree was calculated using PhyML v20160115 (<https://www.genome.jp/tools-bin/clustalw>). % AA identities with VEB-1 are indicated. Red boxes indicate tested variants.

**References**

1.	Naas T, Oueslati S, Bonnin RA et al. Beta-lactamase database (BLDB) - structure and function. *J Enzyme Inhib Med Chem* 2017; **32**: 917-9.
